# Molecular mechanism of PD-1/PD-L1 blockade via anti-PD-L1 antibodies atezolizumab and durvalumab

**DOI:** 10.1038/s41598-017-06002-8

**Published:** 2017-07-17

**Authors:** Hyun Tae Lee, Ju Yeon Lee, Heejin Lim, Sang Hyung Lee, Yu Jeong Moon, Hyo Jeong Pyo, Seong Eon Ryu, Woori Shin, Yong-Seok Heo

**Affiliations:** 1 Department of Chemistry, Konkuk University, 120 Neungdong-ro, Gwangjin-gu, Seoul 05029 Republic of Korea; 2 Department of Bio Engineering, Hanyang University, 222 Wangsimni-ro, Seongdong-gu, Seoul 04763 Republic of Korea

## Abstract

In 2016 and 2017, monoclonal antibodies targeting PD-L1, including atezolizumab, durvalumab, and avelumab, were approved by the FDA for the treatment of multiple advanced cancers. And many other anti-PD-L1 antibodies are under clinical trials. Recently, the crystal structures of PD-L1 in complex with BMS-936559 and avelumab have been determined, revealing details of the antigen-antibody interactions. However, it is still unknown how atezolizumab and durvalumab specifically recognize PD-L1, although this is important for investigating novel binding sites on PD-L1 targeted by other therapeutic antibodies for the design and improvement of anti-PD-L1 agents. Here, we report the crystal structures of PD-L1 in complex with atezolizumab and durvalumab to elucidate the precise epitopes involved and the structural basis for PD-1/PD-L1 blockade by these antibodies. A comprehensive comparison of PD-L1 interactions with anti-PD-L1 antibodies provides a better understanding of the mechanism of PD-L1 blockade as well as new insights into the rational design of improved anti-PD-L1 therapeutics.

## Introduction

Programmed death 1 (PD-1) and its ligands PD-L1 and PD-L2 are key co-inhibitory molecules in the modulation of T-cell mediated immune responses^1–3^. PD-1 is a type I membrane protein with a single extracellular immunoglobulin superfamily (IgSF) V-set domain that is expressed on the surface of activated T cells in peripheral tissues^4, 5^. PD-L1 and PD-L2 are commonly expressed on dendritic cells and macrophages, and their ectodomains are composed of a membrane distal IgSF V-set and a membrane proximal IgSF C-set domains^6, 7^. Ligation of PD-1 with its two ligands initiates co-inhibitory signaling through the cytoplasmic domain of PD-1, containing an immunoreceptor tyrosine-based inhibitory motif and an immunoreceptor tyrosine-based switch motif, thus leading to activation of SHP phosphatases that downregulate TCR signaling by dephosphorylating effector molecules involved in the signaling^8, 9^. As a result, PD-1 signaling prevents excessive or harmful inflammation and maintains immune tolerance to self-antigens under normal conditions^10^.

PD-L1 is often overexpressed in different tumors, and its interaction with PD-1 on T cells enables cancer cells to evade T-cell-mediated immune responses^11^. Thus, blocking the PD-1/PD-L1 interaction can restore T-cell activation and antitumor responses^12–14^. The success of antibody-based PD-1/PD-L1 blockade therapies has provided a major breakthrough in the fight against human cancers, especially for solid tumors. The FDA approved the first anti-PD-L1 drug, atezolizumab (Tecentriq®), for the treatment of advanced urothelial carcinoma in May 2016 and metastatic non-small cell lung cancer (NSCLC) in October 2016^15–18^. In 2017, avelumab (Bavencio®) and durvalumab (Imfinzi®) were also approved by the FDA for Merkel-cell carcinoma and advanced bladder cancer, respectively (Supplementary Table S1). Prior to the approval of the anti-PD-L1 antibodies, the anti-PD-1 drugs pembrolizumab (Keytruda®) and nivolumab (Opdivo®) have been widely used clinically and demonstrated unprecedented therapeutic benefits since 2014^19–23^. In addition, other anti-PD-L1 antibodies including BMS-936559 are in multiple clinical trials for the treatment of NSCLC, renal cell cancer, head and neck cancer, gastric cancer, and other cancers^24–27^.

Combinations of anti-PD-1/PD-L1 antibodies with other therapies are being explored as potentially synergistic therapeutic strategies. Among these, the combination of anti-PD-1/PD-L1 and anti-CTLA-4 antibodies has been the most successful, probably because PD-1/PD-L1 and CTLA-4/B7 interactions play different roles in T-cell immunity^6, 28–30^. A clinical trial of a combination therapy involving durvalumab and an anti-CTLA-4 antibody, tremelimumab, showed antitumor activity in NSCLC regardless of PD-L1 status, suggesting that this combination has therapeutic potential for patients with PD-L1-negative tumors^31^. As PD-L1 is also expressed on activated T cells, the Fc domains of atezolizumab and durvalumab were engineered to eliminate antibody-dependent cellular cytotoxicity (ADCC) or complement-dependent cytotoxicity (CDC), thereby preventing the depletion of T cells expressing PD-L1^32, 33^.

The structures of murine PD-1 in complex with human PD-L1, murine PD-1 in complex with murine PD-L2, and human PD-1 in complex with human PD-L1 have established the structural foundations of the interaction of PD-1 with its ligands, PD-L1 and PD-L2^34–37^. Recently, the crystal structures of PD-L1 in complex with the Fab fragment of BMS-936559 and the single-chain Fv fragment (scFv) of avelumab have been determined, revealing details of the antigen-antibody interactions^38, 39^. However, the epitopes and the PD-L1 blocking mechanism of atezolizumab and durvalumab remain unclear. It is also unknown whether there are novel binding sites on PD-L1 for the design and improvement of anti-PD-L1 agents and whether atezolizumab and durvalumab utilize a similar or distinct competitive binding mode to PD-L1 compared with other anti-PD-L1 antibodies for the development of better combination therapies.

In the present study, we report the crystal structures of the N-terminal IgSF V-set domain of PD-L1 in complex with the Fab fragments of atezolizumab and durvalumab, thereby elucidating the structural basis for the blockade of the PD-1/PD-L1 interaction by these therapeutic antibodies. In addition, a comprehensive analysis of the PD-L1 interactions with the receptor PD-1 and anti-PD-L1 antibodies, including atezolizumab, durvalumab, BMS-963559, and avelumab, provides new insights into the development of future anti-PD-L1 therapeutics, including small-molecule modulators and next-generation therapeutic antibodies.

## Results

### Structure determination of PD-L1 in complex with atezolizumab and durvalumab

**Figure 1:**
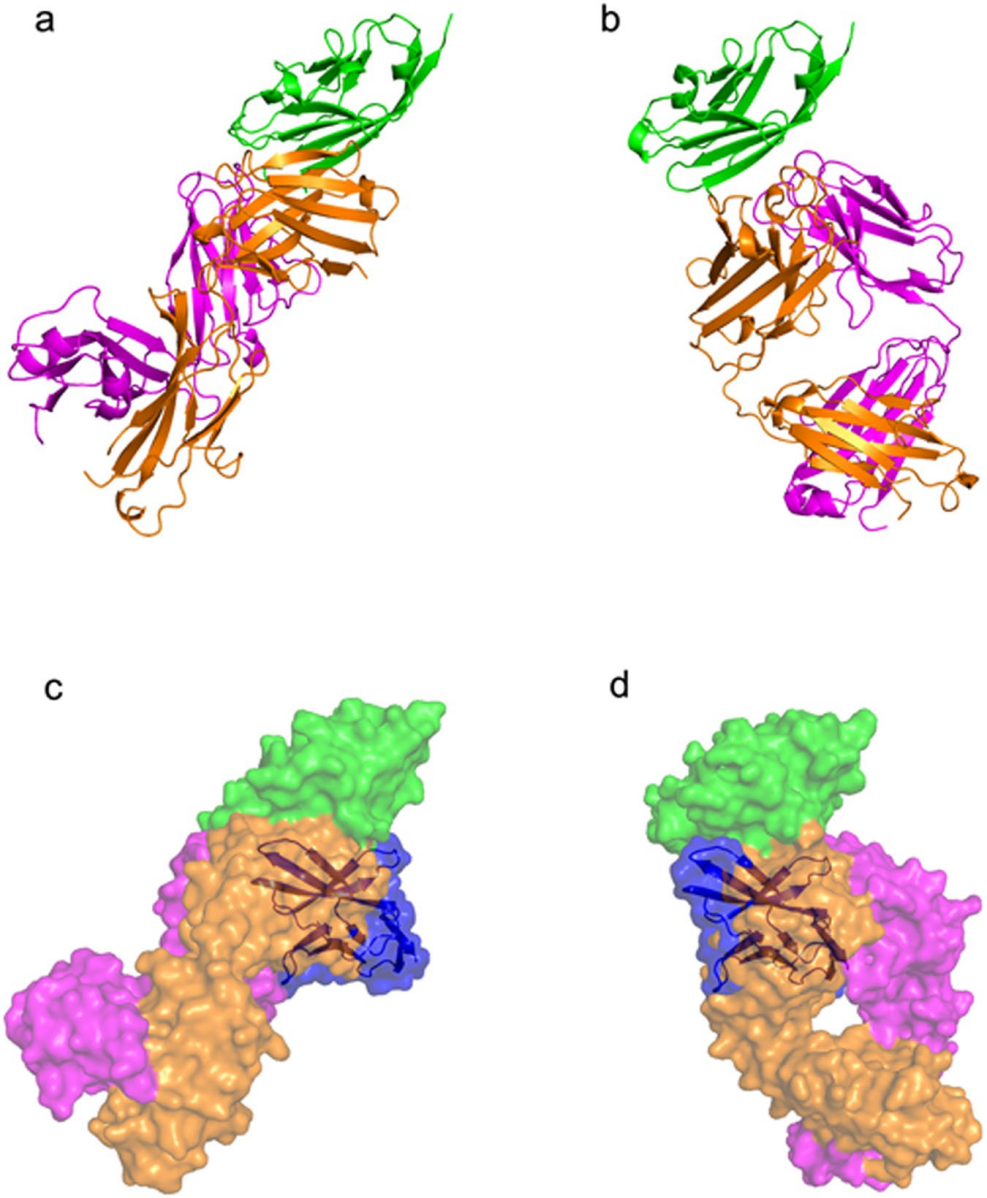
Crystal structures of PD-1 in complex with atezolizumab and durvalumab. (**a**) Ribbon representation of the complex structure of PD-L1/atezolizumab Fab fragment. (**b**) Ribbon representation of the complex structure of PD-L1/durvalumab Fab fragment. (**c**) Surface representation of the complex structure of PD-L1/atezolizumab Fab fragment. (**d**) Surface representation of the complex structure of PD-L1/durvalumab Fab fragment. PD-L1 and the antibody heavy and light chains are colored green, orange, and purple, respectively. In **a**–**d**, PD-L1 is in the same orientation. In **c** and **d**, the PD-1/PD-L1 complex (PDB code 4zqk) is superimposed onto the PD-L1 molecule in the PD-L1/anti-PD-L1 complexes with mixed ribbon/surface representation. PD-1 in the PD-1/PD-L1 complex are colored blue.

**Figure 2:**
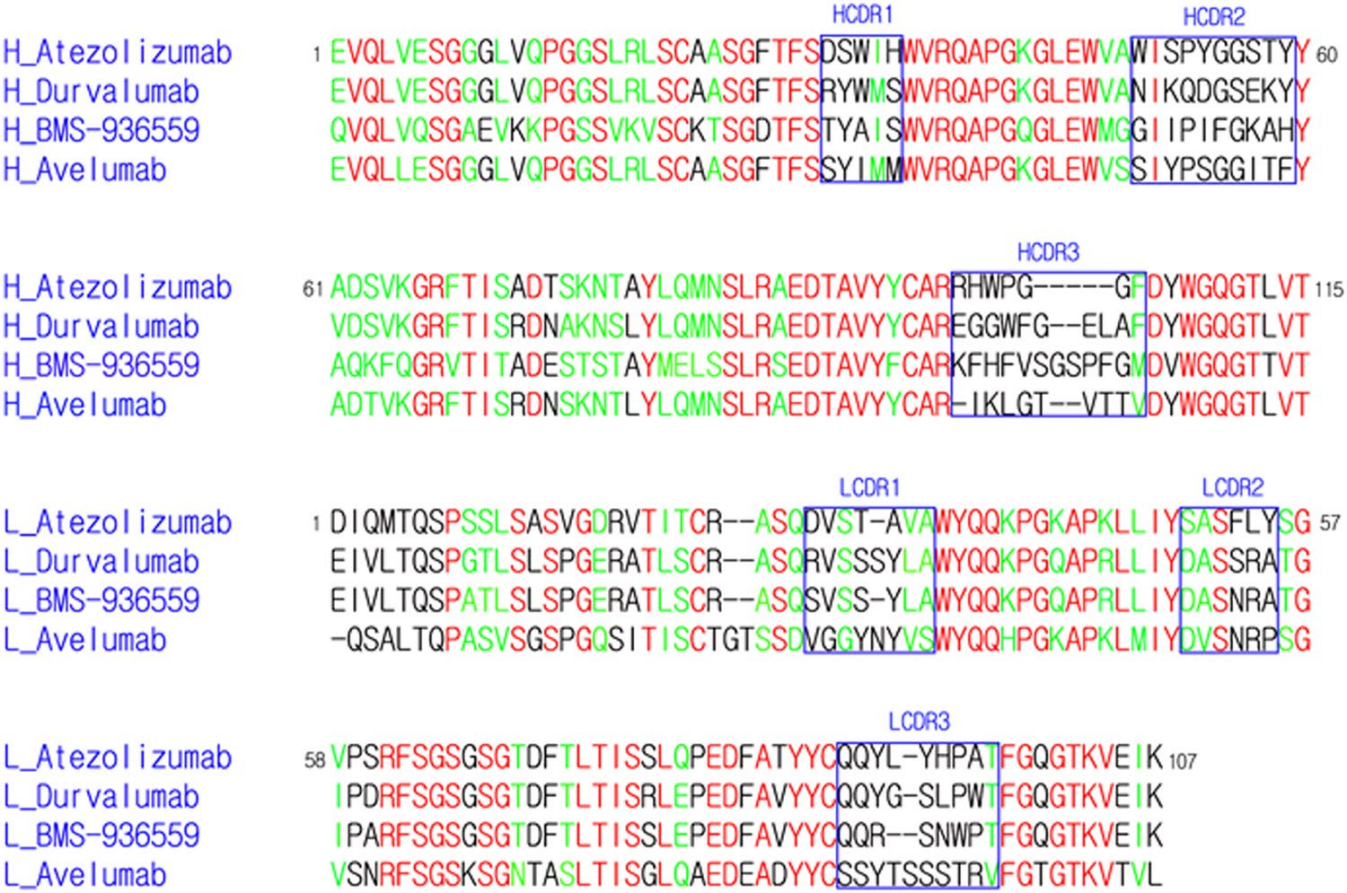
Sequence comparison of the antibodies against PD-L1. The CDRs are indicated with boxes and labeled. The residue numbers refer to those in atezolizumab. The identical and homologous residues are colored red and green, respectively.

In this study, the IgSF V-set domain of PD-L1 was expressed as inclusion bodies in *E*. *coli* and refolded *in vitro* to obtain a soluble form. The Fab fragments of the anti-PD-L1 antibodies were produced by periplasmic expression in *E*. *coli*. Gel filtration analysis confirmed that that the 1:1 complexes of PD-L1 with each antibody exist as monomers in solution. The crystal structure of the IgSF V-set domain of human PD-L1 in complex with the atezolizumab Fab fragment was determined at a resolution of 3.10 Å with R/R_free_ = 0.208/0.256 (Fig. 1a,c). PD-L1 and the atezolizumab Fab form a 1:1 complex in the crystal as in solution, and the crystallographic asymmetric unit contained five copies of the complex without any symmetric relationship among them (Supplementary Figure S1a). Superposition of the PD-L1 protein from the five copies in an asymmetric unit showed that the PD-L1 protein and the variable region of atezolizumab exhibited little structural deviation from each other, whereas the constant region swings almost 30° due to the intrinsic flexibility of the Fab elbow (Supplementary Figure S1b). Almost all residues in the complex, except for a few residues within the loops of the constant region of the atezolizumab Fab fragment, were well defined in the electron density map, clarifying the precise antigen-antibody interactions despite the relatively low resolution of the structural data (Supplementary Figure S2a). We also determined and refined the crystal structure of the IgSF V-set domain of human PD-L1 in complex with the durvalumab Fab fragment at a resolution of 2.65 Å with R/R_free_ = 0.181/0.219 (Fig. 1b,d). The asymmetric unit contained only one PD-L1/durvalumab complex with 1:1 stoichiometry, and all residues in the complex were well defined by the electron density (Supplementary Figure S2b). Both atezolizumab and durvalumab use all three complementarity determining regions (CDRs) from the heavy chain (HCDR1, HCDR2, and HCDR3) and two from the light chain (LCDR1 and LCDR3) to form contacts with PD-L1 (Fig. 2). This is consistent with the general observation that the LCDR2 of antibodies is often not involved in antigen binding. The previously reported structures of PD-L1 in complex with BMS-963559 or avelumab demonstrated that these two antibodies similary involved only five of the six CDRs in the interaction with PD-L1, leaving LCDR2 without any binding to PD-L1^38, 39^.

### Interactions between PD-L1 and atezolizumab

**Figure 3:**
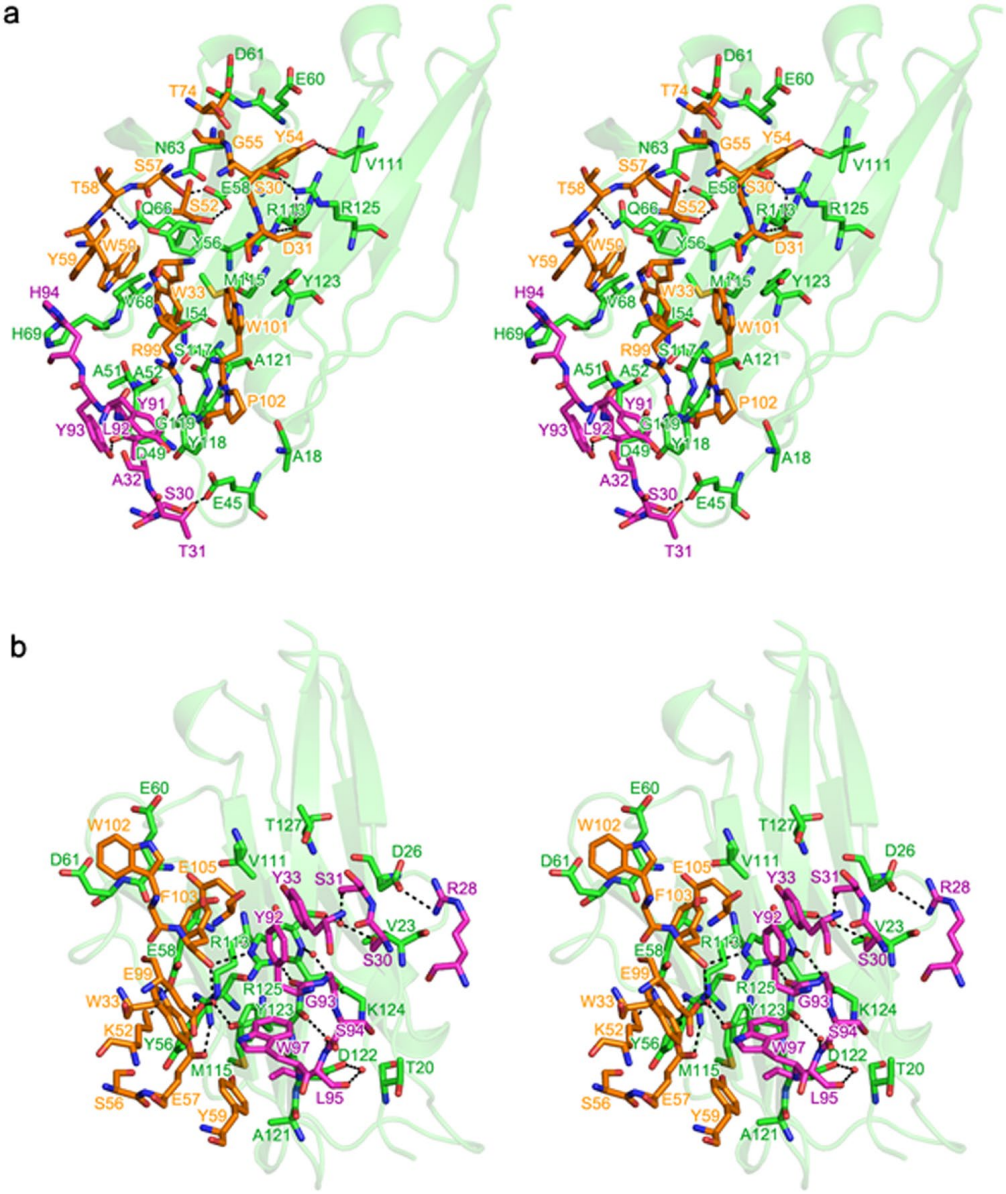
Interactions of atezolizumab and durvalumab with PD-L1. (**a**) Stereoview of the detailed PD-L1/atezolizumab Fab fragment interface. (**b**) Stereoview of the detailed PD-L1/durvalumab Fab fragment interface. In (**a**,**b**) the carbon atoms from PD-L1 and the antibody heavy and light chains are colored green, orange, and purple, respectively. Hydrogen bonds and salt bridges are indicated with dashed lines.

**Figure 4:**
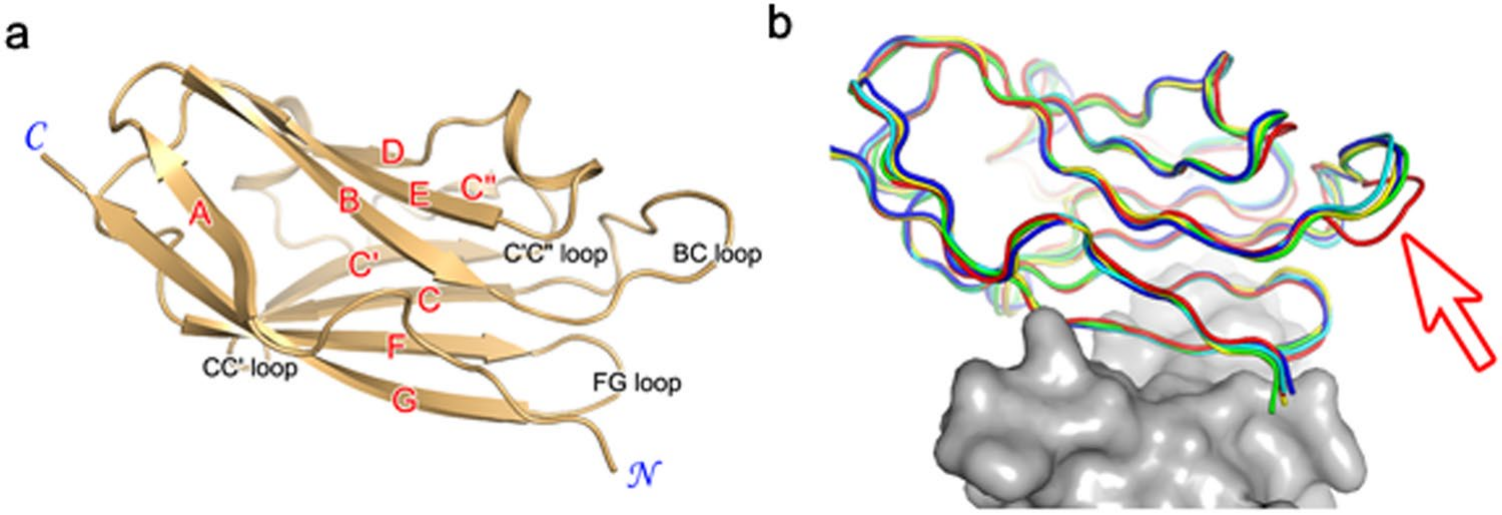
Intrinsic structural rigidity of PD-L1. (**a**) Canonical designation of the strands (red labels) and loops (black labels) within PD-L1. The N- and C-termini are labeled in blue. (**b**) Superposition of the PD-L1 protein extracted from the structures in complex with PD-L1 (blue), atezolizumab (red), durvalumab (cyan), BMS-963559 (yellow), and avelumab (green). The arrow indicates the conformational change of the BC loop in PD-L1 upon binding to atezolizumab. The bound PD-1 protein is shown as a gray surface model. The orientation of PD-L1 is the same as that in (**a**).

The interaction between atezolizumab and PD-L1 buries a total solvent accessible area of 2,106 Å^2^, which is larger than the PD-1/PD-L1 interface (1,970 Å^2^). Most of the buried solvent accessible area is contributed by the heavy chain (67%) (Fig. 3a). The atezolizumab epitope is formed on the surface of PD-L1 by the CC′FG antiparallel β-sheet and the BC, CC′, C′C″, and FG loops (Fig. 4a). In total, 23 residues of PD-L1 participate in the interaction with atezolizumab, forming nine hydrogen bonds, two salt bridges, and extensive van der Waals contacts (Supplementary Table S2). Of course, there should be water-mediated hydrogen bonds within the interface of the antigen-antibody pair, but these cannot be visualized due to the low resolution of 3.10 Å. The complex structure demonstrates that the interaction between PD-L1 and atezolizumab is mediated largely by residues within the central CC′FG β-sheet of PD-L1 and the heavy chain of atezolizumab. The side chain of _PD-L1_E58 makes two hydrogen bonds with the hydroxyl groups of _heavy_S52 and _heavy_S57. The side chain nitrogen atom of _PD-L1_Q66 and the backbone amide group of _PD-L1_V111 form hydrogen bonds with the backbone carbonyl group of _heavy_T58 and the side chain of _heavy_Y54, respectively. _PD-L1_R113 and _PD-L1_R125 make salt-bridge interactions with the side chain of _heavy_D31 and hydrogen bonds with the backbone carbonyl group of _heavy_D31 and the hydroxyl group of _heavy_S30, simultaneously. The side chains of _PD-L1_I54, _PD-L1_Y56, _PD-L1_N63, _PD-L1_V111, _PD-L1_M115, _PD-L1_S117, _PD-L1_A121, and _PD-L1_Y123 in the CC′FG sheet of PD-L1 make van der Waals contacts with the residues within the HCDRs of atezolizumab, including _heavy_D31, _heavy_W33, _heavy_W50, _heavy_Y54, _heavy_S57, and _heavy_W101, through alkyl-alkyl, alkyl-π, and T-shaped stacking interactions. In addition to the interaction mediated by residues of the central CC′FG sheet, residues in the BC, CC′, C′C″, and FG loops also contribute to the interaction with atezolizumab. The side chains of _PD-L1_E45 and _PD-L1_D49 in the BC loop make hydrogen bonds with the side chains of _light_S30 and _light_Y93, respectively. A hydrophobic interaction is also formed between the BC loop and LCDR3, involving _PD-L1_A51 and _PD-L1_A52 in the BC loop and _light_L92 and _light_Y93 in atezolizumab. These interactions draw the BC loop toward the antigen-antibody interface, thereby inducing a conformational change in the BC loop of PD-L1 that deviates from its conformation within PD-L1 in apo form or in complex with PD-1 and other anti-PD-L1 antibodies (Fig. 4b)^34, 38, 39^. _PD-L1_E60 and _PD-L1_D61 within the CC′ loop make van der Waals contacts with _heavy_Y54, _heavy_G55, and _heavy_T74. _PD-L1_V68 and _PD-L1_H69 in the C′C″ loop also contact _heavy_W50, _heavy_Y59, and _light_H94 through alkyl-π and T-shaped stacking interactions. The residues in the FG loop, including _PD-L1_Y118 and _PD-L1_G119, produce hydrophobic interactions with the side chains of _light_T31, _light_A32, _light_Y91, _light_L92, and _heavy_W33, as well as a hydrogen bond between the backbone amide group of _PD-L1_G119 and the side chain of _heavy_R99. Additionally, _PD-L1_A18 in the N-terminus of PD-L1 has a van der Waals interaction with _heavy_P102.

### Interactions between PD-L1 and durvalumab

The interaction between durvalumab and PD-L1 buries a total solvent accessible area of 1,624 Å^2^, which is smaller than the PD-1/PD-L1 interface by 346 Å^2^. The binding interface is almost equally contributed by both the heavy and light chains (Fig. 3b). The durvalumab epitope is constituted by the C strand, F strand, G strand, CC′ loop, and N-terminal region of PD-L1 (Fig. 4a). In total, 16 residues of PD-L1 participate in the interaction with durvalumab through hydrogen bonds, salt bridges, and hydrophobic interactions (Supplementary Table S3). Fortunately, the resolution of the structure enabled the visualization of five water molecules mediating hydrogen bonds in the binding interface between PD-L1 and durvalumab. Most of the key interactions of PD-L1 with durvalumab are concentrated on the central CC′FG β-sheet within PD-L1. The side chain of _PD-L1_R113 makes a bidentate salt bridge with the side chain of _heavy_E57. _PD-L1_E58 also forms an ionic interaction with _heavy_K52. The side chain of _PD-L1_R125 makes two hydrogen bonds with the backbone carbonyl groups of _heavy_F103 and _light_Y92. Five water molecules mediating hydrogen bonds are involving three residues within the G strand, including _PD-L1_D122, _PD-L1_Y123, and _PD-L1_R125. A water molecule creates hydrogen bonds with the side chain of _PD-L1_D122 and the backbone carbonyl group of _light_L95, enabling them to interact. The side chains of _PD-L1_Y123 and _heavy_E99 and the backbone carbonyl group of _heavy_F103 are also connected via water-mediated hydrogen bonds. Two water molecules are located between the backbone atoms of _PD-L1_Y123 and _light_L95 and between _PD-L1_R125 and _light_G93, mediating hydrogen bonds between them. The hydroxyl groups of _light_S30 and _light_S31 interact concurrently with the backbone carbonyl group of _PD-L1_R125 using a water molecule. In addition to the polar interactions, the side chains of _PD-L1_Y56, _PD-L1_V111, _PD-L1_M115, _PD-L1_A121, _PD-L1_Y123, _PD-L1_K124, and _PD-L1_T127 in the CC′FG β-sheet of PD-L1 make van der Waals contacts with the residues in the CDRs of durvalumab, including _heavy_W33, _heavy_S56, _heavy_Y59, _heavy_F103, _heavy_E105, _light_Y33, _light_S94, _light_L95, and _light_W97. Besides the main interaction by the residues within the CC′FG sheet, the CC′ loop and N-terminal region of PD-L1 also contribute to the interaction with durvalumab. The bulky side chain of _heavy_W102 in durvalumab makes van der Waals contacts with _PD-L1_E60 and _PD-L1_D61 within the CC′ loop of PD-L1. The N-terminal region of PD-L1 also participates in the interaction with durvalumab through an ionic interaction of _PD-L1_D26 with _light_R28 and van der Waals contacts by _PD-L1_T20 and _PD-L1_V23.

### Comparison of the interfaces between PD-L1 and PD-1 and anti-PD-L1 antibodies

**Figure 5:**
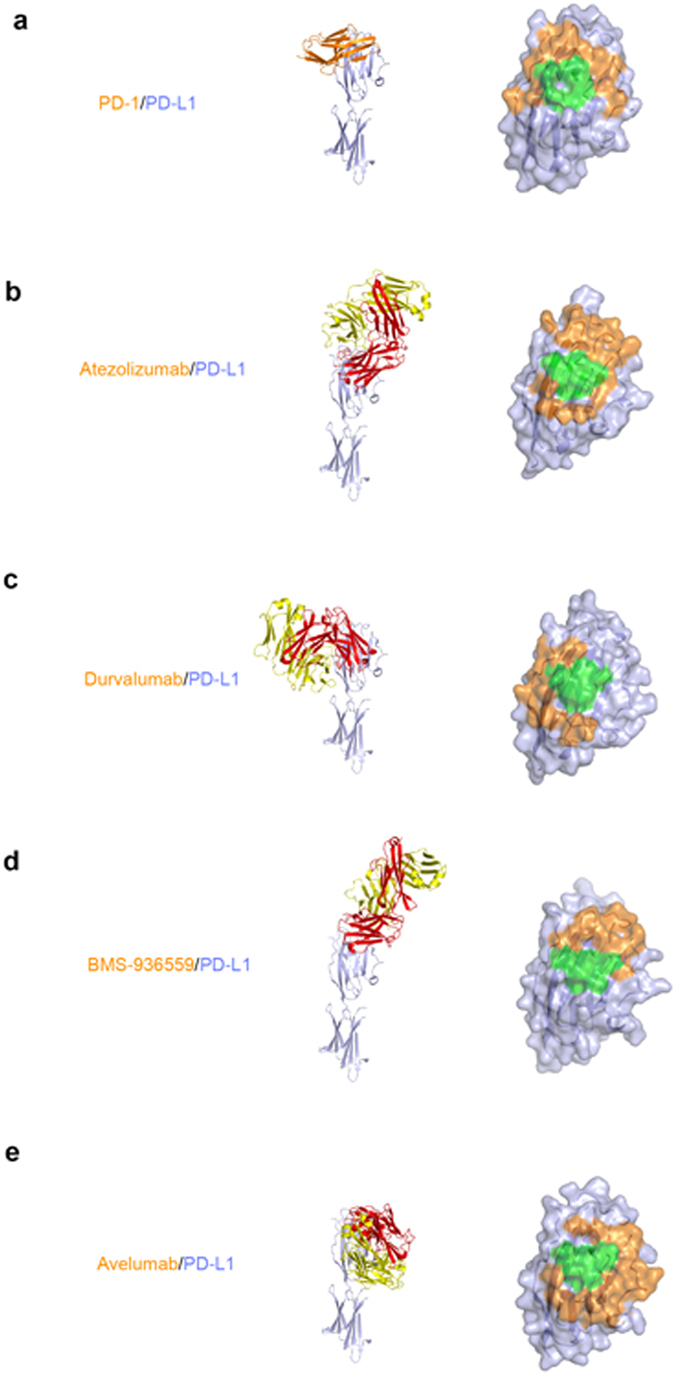
Comparison of the PD-L1 interactions with the receptor PD-1 and anti-PD-L1 antibodies. (**a**) Structure of PD-L1 (pale blue) in complex with PD-1 (orange) and the PD-1 binding site (orange) on the surface of the IgSF V-set domain of PD-L1. (**b**) Structure of PD-L1 in complex with atezolizumab Fab and its epitope on the surface of the IgSF V-set domain of PD-L1. (**c**) Structure of PD-L1 in complex with durvalumab Fab and its epitope on the surface of the IgSF V-set domain of PD-L1. (**d**) Structure of PD-L1 in complex with BMS-963559 Fab and its epitope on the surface of the IgSF V-set domain of PD-L1. (**e**) Structure of PD-L1 in complex with avelumab Fab and its epitope on the surface of the IgSF V-set domain of PD-L1. In (**a**–**e**) the IgSF V-set and IgSF C-set domains of PD-L1 are displayed in the same orientation, and the antibody heavy and light chains and their epitopes are colored red, yellow, and orange, respectively. The shared regions on the epitopes of the four antibodies and the PD-1 binding site are colored green.

A comprehensive comparison of the PD-L1 interactions with the receptor PD-1 and anti-PD-L1 antibodies, including atezolizumab, durvalumab, BMS-963559, and avelumab, can provide a better understanding of the mechanism of PD-L1 blockade by these therapeutic antibodies as well as new insights into the rational design of improved anti-PD-L1 therapeutics (Fig. 5)^34, 37–39^.

The antibodies bind to PD-L1 from various directions and with different binding sites. Atezolizumab and BMS-963559 bind to the upper side close to the N-terminus of PD-L1, heavily tilted toward the face containing the central CC′FG β-sheet, implying the IgG form of these antibodies would take a narrow Y-shaped form when binding two neighboring PD-L1 molecules with two arms of IgG (Fig. 5b,d). In contrast, durvalumab and avelumab bind rather perpendicularly to PD-L1; therefore, the bivalent IgG of these antibodies would be T-shaped rather than Y-shaped when binding to two PD-L1 molecules at the same time (Fig. 5c,e). The dissociation constant (K_d_) for the binding of avelumab-scFv to PD-L1 has been reported to be 42.1 pM by surface plasmon resonance (SPR) and other antibodies are also known to have K_d_ values less than 1 nM, whereas the binding affinity between PD-1 and PD-L1 was reported as 8.2 μM by SPR^35, 39–42^. The buried surface areas of PD-L1 in complex with PD-1, atezolizumab, durvalumab, BMS-963559, and avelumab are 1970, 2106, 1624, 1349, and 1865 Å^2^, respectively (Fig. 5). Simple comparison of these values cannot explain the large gap in the PD-L1 binding affinity between PD-1 and anti-PD-L1 antibodies. Considering the previous report that directed evolution on the binding surface of PD-1 by yeast-surface display generated a high-affinity PD-1 mutant (110 pM) that antagonizes PD-L1 competitively, the low affinity of PD-1 is probably due to the incomplete complementarity of the interface between PD-1 and PD-L1^43^. The low affinity of the PD-1/PD-L1 pair should be more suitable for the transient interaction to modulate T-cell mediated immune responses in a timely manner through reversible interactions.

Although each antibody elicits a different epitope from the others, the central CC′FG β-sheet within PD-L1 provides key interactions for binding to all the antibodies (Fig. 5). The shared region on the epitopes of the four antibodies includes _PD-L1_Y56, _PD-L1_E58, _PD-L1_R113, _PD-L1_M115, and _PD-L1_Y123, which are located within the CC′FG β-sheet and provide pivotal interactions when PD-L1 binds to PD-1. Therefore, the overlap of the epitopes within the surface of the CC′FG sheet implies that the mechanism by which the anti-PD-L1 antibodies block the PD-1/PD-L1 interaction is by outcompeting PD-1 for binding to PD-L1. This is due to the much higher affinity of the antibodies and the increased avidity from the bivalency of IgG. In addition to the predominant interaction mediated by the residues of the central CC′FG β-sheet, the loops within PD-L1 are also involved in the interaction with each antibody, contributing to the stabilization of the antigen-antibody complexes. As described above, the BC, CC′, C′C″, and FG loops of PD-L1 make extensive interactions with atezolizumab, and the CC′ loop and N-terminal region are also involved in the interaction with durvalumab through a salt bridge and van der Waals contacts. The structure of PD-L1 in complex with BMS-963559 showed that the BC, C′C″, and FG loops provided key interactions, and the PD-L1-avelumab complex structure demonstrated that the CC′ loop contributed to a major interaction with avelumab through multiple hydrogen bonds provided by _PD-L1_D61^38, 39^. Taken together, the BC, CC′, C′C″, and FG loops as well as the CC′FG sheet could be vulnerable antigenic sites for anti-PD-L1 therapeutic antibodies. These loops may also be valuable hot-spots for the design of small-molecule modulators of the PD-1/PD-L1 interaction. As the surface of the central CC′FG β-sheet is flat, it is very difficult to design a small molecule with a high binding affinity by targeting only this region. Additional interactions with the loops would be critical to acquiring a high potency against PD-L1. Analysis of the diverse interactions of these loops with the antibodies should enable us to design promising small-molecule PD-1/PD-L1 blockers, which can overcome the drawbacks of antibody-based therapeutics.

## Discussion

Cancer immunotherapy via antibody-based PD-1/PD-L1 blockade has provided a major breakthrough for the treatment of multiple advanced and metastatic cancers since the approval of the monoclonal antibodies targeting the PD-1/PD-L1 axis. Despite the great achievement of the therapeutic antibodies blocking the PD-1/PD-L1 interaction, they have specific shortcomings as therapeutics. Poor tissue/tumor penetrance of antibody drugs due to their large size can be problematic, especially when targeting PD-1/PD-L1 signaling as PD-1-expressing T cells are found infiltrated within the solid tissue of PD-L1-expressing tumors^44, 45^. To compensate in part for the possible suboptimal efficacy of therapeutic antibodies, the development of low-molecular weight protein drugs or small molecules modulating PD-1/PD-L1 signaling is urgently needed, and a combination therapy of small-molecule modulators and antibody drugs may be an excellent option for the treatment of cancers through complete PD-1/PD-L1 blockade in solid tumors. Directed evolution based on the crystal structure of PD-1/PD-L1 complex enabled to engineer the PD-1 ectodomain as a high-affinity (110 pM) competitive antagonist of PD-1 showing superior tumor penetration^43^. There are also several small-molecule immunomodulators targeting the PD-1/PD-L1 axis in preclinical or clinical investigations^46^. Structural studies on the PD-L1 interaction with therapeutic antibodies can provide insight into the design of small molecules targeting PD-L1, as their potency can be enhanced by mimicking the diverse interactions of these antibodies, including the involvement of the BC, CC′, C′C″, and FG loops for binding to atezolizumab; the CC′ loop and N-terminal region for durvalumab; the BC, C′C″, and FG loops for BMS-963559; and the CC′ loop for avelumab. We also believe that the accumulation of such structural studies will provide invaluable information for developing next-generation therapeutic antibodies, such as antibody drug conjugates (ADCs) and bi-specific antibodies, and for coping with any possible antigen mutational escape of PD-L1 in future.

**Figure 6:**
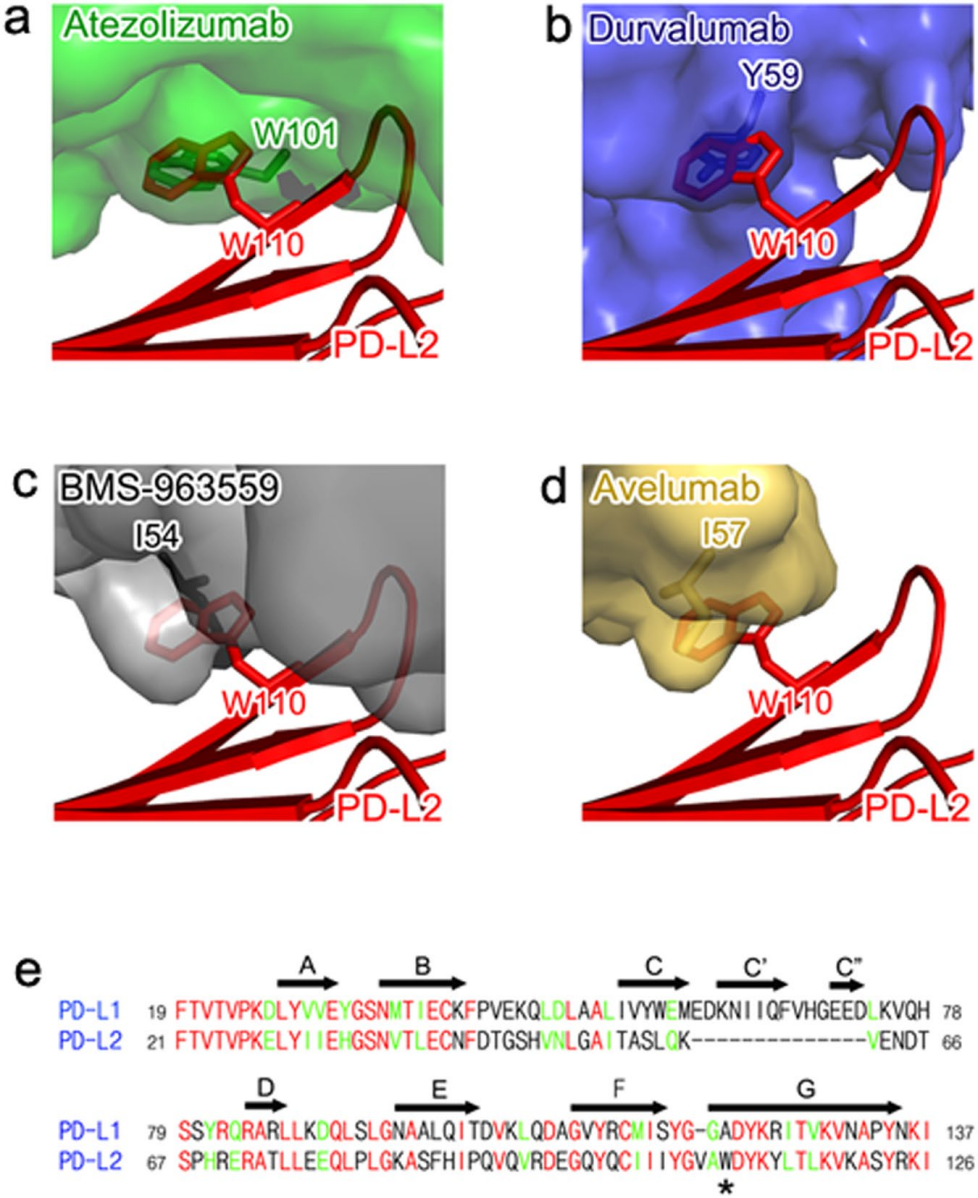
Structural basis for the lack of the binding of anti-PD-L1 antibodies to PD-L2. (**a**) The partially transparent surface model of atezolizumab (green) when PD-L1 of PD-L1/atezolizumab is overlaid onto PD-L2 (red, PDB code 3bov). (**b**) The surface model of durvalumab (blue) when PD-L1 of PD-L1/durvalumab is overlaid onto PD-L2. (**c**) The surface model of BMS-963559 (gray) when PD-L1 of PD-L1/BMS-963559 is overlaid onto PD-L2. (**d**) The surface model of avelumab (yellow) when PD-L1 of PD-L1/avelumab is overlaid onto PD-L2. In (**a**–**d**) the residues of the antibodies, which collide with W110 of PD-L2, are shown in sticks and labeled. (**e**) Structure-based sequence alignment of PD-L1 and PL-L2. The strands in PD-L1 are denoted with arrows above the sequence. W110 of PD-L2 is indicated with an asterisk. The identical and homologous residues are colored red and green, respectively.

PD-L2 also plays a role in maintaining peripheral tolerance in lung via interaction with PD-1 or other receptors such as RGMb^47^. PD-L2-deficient mice exhibit increased airway hyperactivity and lung inflammation^48^. While PD-1 inhibition by anti-PD-1 antibodies disrupts both PD-L1 and PD-L2 pathways, anti-PD-L1 antibodies, including atezolizumab, durvalumab, BMS-963559, and avelumab, are known to target only PD-L1 to inhibit the PD-1/PD-L1 interaction while preserving the PD-1/PD-L2 interaction, thereby avoiding the immune-related toxicity associated with PD-L2 blockade^24^. The complex structures of PD-L1 with these antibodies explain their lack of binding to PD-L2 (Fig. 6). The crystal structure of PD-1 in complex with PD-L2 showed that PD-L1 and PD-L2 have similar binding modes to PD-1^36^. It has been reported that mutation of _PD-L2_W110 to alanine in PD-L2 reduces binding affinity to PD-1 to 40% of that of the wild type^36^. This is probably because _PD-L2_W110, which is located within the G strand of PD-L2, occupies a small hydrophobic pocket on the surface of PD-1, thereby contributing the binding energy for PD-1/PD-L2 interaction. The residue corresponding to _PD-L2_W110 is _PD-L1_A121 in PD-L1, and this difference in the side chain would lead to a 3-fold lower binding affinity of PD-L1 to PD-1 than that of PD-L2. The anti-PD-L1 antibodies contact _PD-L1_A121 with hydrophobic residues, including _heavy_W101 of atezolizumab, _heavy_Y59 of durvalumab, _heavy_I54 of BMS-963559, and _heavy_I57 of avelumab (Fig. 6a–d). The substitution of _PD-L1_A121 with tryptophan should sterically collide with the residues of the anti-PD-L1 antibodies due to its bulky size, thereby leading to failure in binding to PD-L2. In addition, the structure-based sequence alignment of PD-L1 and PD-L2 shows the absence of the CC′ loop, C′ strand, C′C″ loop, and C″ strand in PD-L2 (Fig. 6e). As these regions in PD-L1 provide key interactions for the binding of the antibodies, the absence of them from PD-L2 should also negatively affect the binding of the antibodies to PD-L2.

**Figure 7:**
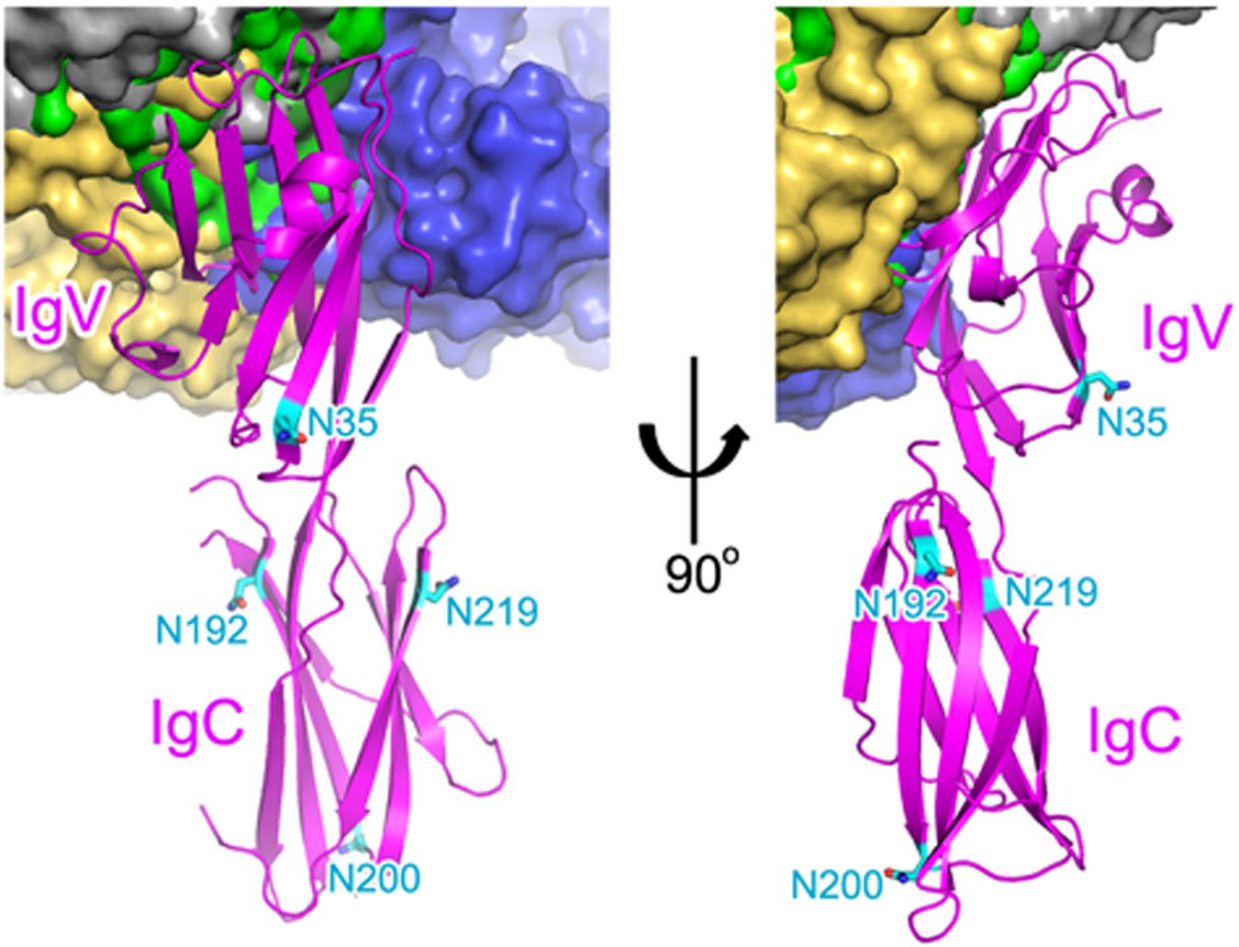
Glycosylation of PD-L1 and antibody binding. The four N-glycosylation sites (N35, N192, N200, and N219) of PD-L1 are shown in sticks and colored cyan. The IgSF V-set and IgSF C-set domains of PD-L1 are labeled. The bound antibodies, including atezolizumab, durvalumab, BMS-963559, and avelumab, are shown as green, blue, gray, and yellow surfaces, respectively.

Protein glycosylation plays a critical role in many biological processes and cancer cells display numerous alterations in glycosylation patterns compared with normal cells, thereby contributing to altered cancer cell functions^49, 50^. As PD-L1 is overexpressed in cancer cells, possible alterations in the glycosylation patterns of PD-L1 would affect the binding of therapeutic antibodies to PD-L1. It has been reported recently that PD-L1 is exclusively N-glycosylated at _PD-L1_N35, _PD-L1_N192, _PD-L1_N200, and _PD-L1_N219 in cancer cells^51^. A structural analysis can estimate the influence of PD-L1 glycosylation on the interaction of the anti-PD-L1 antibodies, including atezolizumab, durvalumab, BMS-963559, and avelumab (Fig. 7). _PD-L1_N35 is located within the B strand, which is on the opposite side of the central CC′FG β-sheet, and _PD-L1_N192, _PD-L1_N200, and _PD-L1_N219 are residues of the IgSF C-set domain, which is not involved in the interaction with these antibodies. Therefore, the binding of these antibodies to PD-L1 should be independent of the glycosylation of PD-L1.

The interaction between PD-1 and PD-L1 is associated with significant plasticity of PD-1. Through the conformational rearrangement within the CC′ loop of PD-1, four additional hydrogen bonds can be formed between PD-1 and PD-L1, thereby contributing to the binding energy of the ligand-receptor interaction^34, 37^. The complex formation-associated plasticity within PD-1 can also be seen in the interaction with the anti-PD-1 antibodies pembrolizumab and nivolumab^38, 52–54^. Binding of these antibodies induces drastic structural changes within the BC, C’D, FG, and N-terminal loops of PD-1, thereby stabilizing the antigen-antibody complexes. In contrast to PD-1, the structure of PD-L1 changes little upon binding to the receptor or to anti-PD-L1 antibodies^34, 37–39^. When binding to PD-1, no significant change within the backbone is induced and only minor adjustments in the arrangement of the side chains occur due to local steric constraints of the binding interface. The CC′, C′C″, and FG loops of PD-L1 are also involved in the interaction with the anti-PD-L1 antibodies including atezolizumab, durvalumab, BMS-963559, and avelumab. However, the binding of PD-L1 to the antibodies does not alter the conformations of these loops in PD-L1, implying that PD-L1 maintains these loops in the productive binding conformation prior to interacting with the receptor or antibodies. Only when binding to atezolizumab does the BC loop of PD-L1 move a little toward the binding interface to make additional interactions with atezolizumab. Compared to the high affinity of the anti-PD-L1 antibodies, the much weaker binding affinity of PD-1 to PD-L1 may be partly based on the intrinsic plasticity of PD-1.

In summary, we reported the crystal structures of the N-terminal IgSF V-set domain of PD-L1 in complex with the Fab fragments of atezolizumab and durvalumab, elucidating the precise epitopes involved and the structural basis for the blockade of the PD-1/PD-L1 interaction by these therapeutic antibodies. A comprehensive analysis of the PD-L1 interactions with the receptor PD-1 and anti-PD-L1 antibodies, including atezolizumab, durvalumab, BMS-963559, and avelumab, demonstrated that the overlap of the epitopes within the surface of CC′FG sheet implies the mechanism of the PD-1/PD-L1 blockade by the therapeutic antibodies. The epitopes and binding modes of the FDA-approved anti-PD-L1 antibodies can be references for the development of other antibodies in future and the BC, CC′, C′C″, and FG loops of PD-L1 should provide key interactions for the development of improved anti-PD-L1 therapeutics including next-generation therapeutic antibodies and small-molecule modulators.

## Methods

### Expression and purification of PD-L1

Genes encoding the IgSF V-set domain of human PD-L1 (aa 18-134) were subcloned into pET-21a (Novagen). The protein was expressed in *E*. *coli* BL21(DE3) as inclusion bodies. The cells were grown at 37 °C in LB medium supplemented with 50 μg mL^−1^ ampicillin until OD_600_ reached 0.6–1.0, and the protein expression was induced with 1 mM IPTG and incubated for 4 h at 37 °C. The cells were harvested by centrifugation, re-suspended in lysis buffer (20 mM Tris, pH 8.0, 200 mM NaCl) and lysed by sonication on ice. Inclusion bodies were recovered by centrifugation (25,000 × g for 0.5 h at 4 °C) and solubilized in 8 M urea, 20 mM Tris, pH 8.0, 200 mM NaCl by stirring overnight. After removing undissolved residue by centrifugation (25,000 × g for 0.5 h at 4 °C), solubilized fraction was applied to HisTrap HP column (GE Healthcare Life Sciences) and washed with five column volumes of wash buffer (8 M urea, 20 mM Tris, pH 8.0, 200 mM NaCl, 50 mM imidazole). The protein was then eluted with elution buffer (8 M urea, 20 mM Tris, pH 8.0, 200 mM NaCl, 400 mM imidazole). The eluted protein was refolded by dialysis 3 times against 20 mM Tris, pH 8.0, 200 mM NaCl and purified further by gel filtration chromatography using a HiLoad 16/60 Superdex 200 pg column (GE Healthcare Life Sciences). The protein purity was evaluated by reducing and nonreducing SDS-PAGE.

### Expression and purification of Fab fragments

The DNA sequences for the Fab fragments of atezolizumab and durvalumab were synthesized after codon-optimization for expression in *E*. *coli* (Bioneer, Inc). The sequences for heavy chain and light chain were cloned into a modified pBAD vector, containing the STII signal sequence in each chain for periplasmic secretion and a C-terminal 6His-tag in heavy chain^38^. The plasmid pBAD-Fab was transformed into *E*. *coli* Top10F (Invitrogen). The cells were grown at 37 °C in LB medium supplemented with 50 μg mL^−1^ ampicillin. At an OD_600_ of 1.0, the protein expression was induced with 0.2% arabinose and cells were grown at 30 °C for 15 h. The cells were harvested by centrifugation, re-suspended in lysis buffer (20 mM Tris, pH 8.0, 200 mM NaCl) and lysed by sonication on ice. After removing cell debris by centrifugation (25,000 × g for 0.5 h at 4 °C), the supernatant containing soluble protein was applied to HisTrap HP column (GE Healthcare Life Sciences) and washed with five column volumes of wash buffer (20 mM Tris, pH 8.0, 300 mM NaCl, 50 mM imidazole). The protein was then eluted with elution buffer (20 mM Tris, pH 8.0, 300 mM NaCl, 400 mM imidazole). The eluted protein was concentrated for gel filtration chromatography using a HiLoad 16/60 Superdex 200 pg column (GE Healthcare Life Sciences). The column had previously been equilibrated with gel filtration buffer (20 mM Tris, pH 8.0, 300 mM NaCl). The elution profile of the protein showed a single major peak and the protein quality was evaluated by reducing and nonreducing SDS-PAGE.

### Crystallization and structure determination

Details of the crystallization, X-ray data collection, structure determination, and refinement of the PD-L1/atezolizumab Fab and PD-L1/durvalumab Fab complexes are described in Supplementary Information. Data collection and refinement statistics are summarized in Table 1.

**Table 1:** Data collection and refinement statistics.

	PD-L1/atezolizumab Fab	PD-L1/durvalumab Fab
Data Collection		
X-ray source	PLS 5C	PLS 7A
Wavelength (Å)	1.0000	1.0000
Space group	*P*2_1_2_1_2_1_	*P*2_1_2_1_2_1_
Cell dimensions		
*a*, *b*, *c* (Å)	92.20, 169.79, 206.10	39.95, 97.39, 153.56
Resolution (Å)	3.10 (3.15–3.10)*	2.65 (2.70–2.65)
*R*_sym_ (%)	11.3 (46.8)	11.0 (43.6)
*I/σI*	6.9 (2.1)	16.9 (3.1)
Completeness (%)	97.0 (96.6)	99.4 (99.1)
Redundancy	3.4 (3.4)	5.2 (5.1)
Refinement		
Resolution (Å)	3.10	2.66
No. reflections	57602	17827
*R*_work_/*R*_free_ (%)	20.8/25.6	18.1/21.9
No. atoms		
Protein	20569	4201
Water	0	73
R.m.s. deviation		
Bond lengths (Å)	0.008	0.008
Bond angles (°)	1.262	1.105
Ramachandran		
Favored (%)	95.16	96.07
Allowed (%)	4.69	3.93
Outlier (%)	0.15	0.00
PDB code	5X8L	5X8M

^*^Values in parentheses are for the outer resolution shell.

### Data availability

The atomic coordinates and structure factors for the structures of PD-L1 in complex with the atezolizumab and durvalumab Fab have been deposited into Protein Data Bank (http://www.rcsb.org) under the accession codes 5X8 L and 5X8 M, respectively.

## Electronic supplementary material

Supplementary Information
