# Supplementary material for: Molecular mechanism of PD-1/PD-L1 blockade via anti-PD-L1 antibodies atezolizumab and durvalumab
Source: Sci Rep. 2017 Jul 17;7:5532. doi: 10.1038/s41598-017-06002-8 (PMC5514103; doi:10.1038/s41598-017-06002-8)
Supplement: Supplementary file 1 — Supplementary Information [file 41598_2017_6002_MOESM1_ESM.pdf]

SUPPLEMENTARY INFORMATION

Molecular mechanism of PD-1/PD-L1 blockade via anti-PD-L1 antibodies atezolizumab and durvalumab

Hyun Tae Lee[1,3], Ju Yeon Lee[1,3], Heejin Lim[1,3], Sang Hyung Lee[1], Yu Jeong Moon[1], Hyo Jeong Pyo[1], Seong Eon Ryu[2], Woori Shin[1] & Yong-Seok Heo[1*]

SUPPLEMENTARY FIGURES

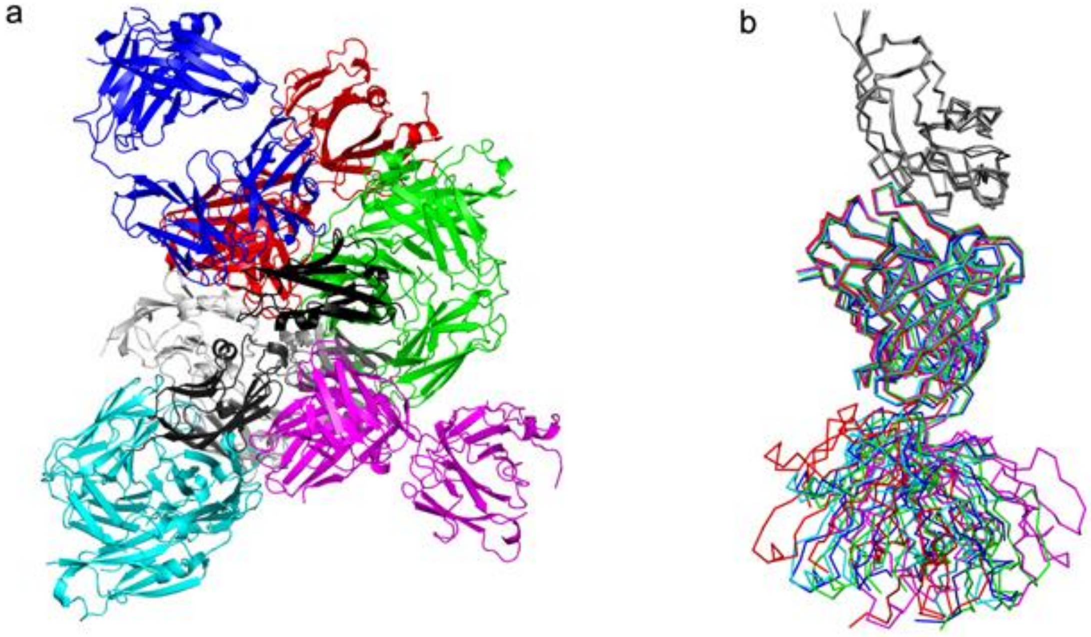

Supplementary Figure S1. Five copies of the PD-L1/atezolizumab Fab complex in an asymmetric unit of crystals.

a. PD-L1 is colored from gray to black and each atezolizumab is a different color.

b. Superposition of the PD-L1 protein from the five copies shows the flexibility within the Fab elbow of atezolizumab.

a

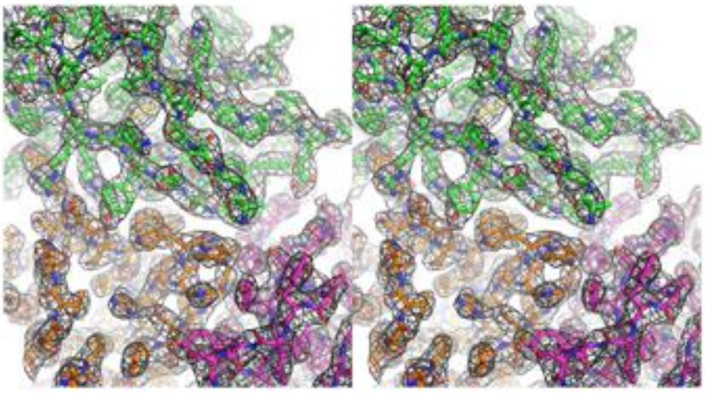

b

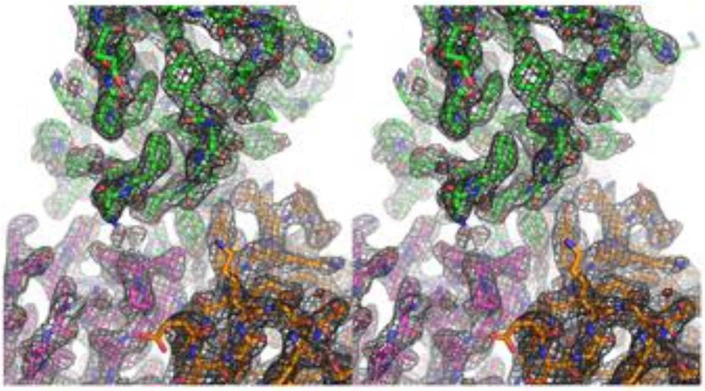

Supplementary Figure S2. Electron densities of the complex structures.

a. Stereoview of the 2fo-fc composite omit map (1.2σ contour level) at the interface between PD-L1 and atezolizumab, calculated at 3.10 Å .

b. Stereoview of the 2fo-fc composite omit map (1.2σ contour level) at the interface between PD-L1 and durvalumab, calculated at 2.65 Å .

In **a** and **b,** the carbon atoms from PD-L1 and the antibody heavy and light chains are colored green, orange, and purple, respectively.

SUPPLEMENTARY TABLES

Supplementary Table S1. Therapeutic antibodies against PD-L1.

**Agent**	**Antibody class**	**Developer**	**Stage of development**
Atezolizumab (Tecentriq®)	Fc modified humanized IgG1κ	Genentech /Roche	FDA approved in 2016
Durvalumab (Imfinzi®)	Fc modified fully human IgG1κ	Medimmune /Astrazeneca	FDA approved in 2017
BMS-936559	Fully human IgG4 κ	Bristol-Myers Squibb	Phase I/II
Avelumab (Bavencio®)	Fully human IgG1κ	Merck Serono /Pfizer	FDA approved in 2017

Supplementary Table S2. Interactions between PD-L1 and atezolizumab.

PD-L1 contact residue	PD-L1 residue location	Atezolizumab contact residue
A18	N-terminus	$_{heavy}$P102
E45	BC loop	$_{light}$S30,
D49	BC loop	$_{light}$Y93
A51	BC loop	$_{light}$Y93
A52	BC loop	$_{light}$L92, $_{light}$Y93
I54	C strand	$_{heavy}$W33, $_{heavy}$W50
Y56	C strand	$_{heavy}$W33, $_{heavy}$W50, $_{heavy}$S57
E58	C strand	$_{heavy}$S52, $_{heavy}$S57
E60	CC´ loop	$_{heavy}$Y54, $_{heavy}$T74
D61	CC´ loop	$_{heavy}$ G55, $_{heavy}$T74
N63	C´ strand	$_{heavy}$S57
Q66	C´ strand	$_{heavy}$T58
V68	C´C´´ loop	$_{heavy}$W50, $_{heavy}$Y59
H69	C´C´´ loop	$_{heavy}$Y59, $_{light}$H94
V111	F strand	$_{heavy}$Y54
R113	F strand	$_{heavy}$D31
M115	F strand	$_{heavy}$W33, $_{heavy}$W50, $_{heavy}$W101
S117	F strand	$_{heavy}$W33
Y118	FG loop	$_{light}$T31, $_{light}$L92
G119	FG loop	$_{light}$A32, $_{light}$Y91, $_{heavy}$R99
A121	G strand	$_{heavy}$W33, $_{heavy}$W101
Y123	G strand	$_{heavy}$W101, $_{heavy}$D31
R125	G strand	$_{heavy}$S30, $_{heavy}$D31

Supplementary Table S3. Interactions between PD-L1 and durvalumab.

**PD-L1 contact residue**	**PD-L1 residue location**	**Durvalumab contact residue**
T20	N-terminus	$_{light}$S94, $_{light}$L95
V23	N-terminus	$_{light}$S30
D26	N-terminus	$_{light}$R28
Y56	C strand	$_{heavy}$S56
E58	C strand	$_{heavy}$K52
E60	CC´ loop	$_{heavy}$W102
D61	CC´ loop	$_{heavy}$W102
V111	F strand	$_{light}$Y33, $_{heavy}$F103, $_{heavy}$E105
R113	F strand	$_{heavy}$E57
M115	F strand	$_{heavy}$Y59
A121	G strand	$_{heavy}$Y59
D122	G strand	$_{light}$L95
Y123	G strand	$_{light}$L95, $_{light}$W97, $_{heavy}$W33, $_{heavy}$E99, $_{heavy}$F103
K124	G strand	$_{light}$S94
R125	G strand	$_{light}$S30, $_{light}$S31, $_{light}$Y92, $_{light}$G93, $_{heavy}$F103
T127	G strand	$_{heavy}$W33

SUPPLEMENTARY METHODS

Crystallization and structure determination of PD-L1/atezolizumab Fab complex

Purified PD-L1 and atezolizumab Fab fragment were mixed in 1:1 molar ratio and incubated for 1 h at 4 ℃ before being subjected to size exclusion chromatography using a HiLoad 16/60 Superdex 200 pg column (GE Healthcare) equilibrated with 20 mM Tris, pH 8.0, and 300 mM NaCl. Gel-filtration fractions containing the PD-L1/atezolizumab Fab complex was concentrated to 6 mg ml^{-1} in 20 mM Tris, pH 8.0, and 300 mM NaCl. Crystals of the complex were grown using hanging-drop vapor diffusion with a reservoir solution containing 0.1 M sodium HEPES, pH 7.5, 20% PEG8000 at 20 ℃ within a month. Crystals were cryoprotected by brief immersion in well solution supplemented with 25% glycerol, and flash frozen in liquid nitrogen. X-ray diffraction data were collected at 100 K on beamline 5C of the Pohang Light Source (PLS), Republic of Korea. The crystals belonged to space group $P2_12_12_1$ (a = 92.20, b = 169.79, c = 206.10 Å) and X-ray diffraction data were collected to a resolution of 3.10 Å , integrated and scaled using HKL2000 (HKL Research)[1]. The structure was solved by molecular replacement using Phaser[2] with the separate structures of the Fab fragment and PD-L1 in the structure of PD-L1/BMS-936559 Fab complex (PDB code 5GGT) as search models. Due to the intrinsic elbow flexibility of a Fab fragment, the Fv region and the other region including C_{H1} and C_L domain were separated when used as a search model. At this point, the electron density corresponding the PD-L1/atezolizumab Fab complex was prominent. Iterative rounds of refinement were done using PHENIX[3] with manual inspection using COOT[4]. Statistics for data collection and refinement can be found in Table 1. All structural figures were generated using Pymol (http://www.pymol.org).

Crystallization and structure determination of PD-L1/durvalumab Fab complex

Purified PD-L1 and durvalumab Fab fragment were mixed in 1:1 molar ratio and incubated for 1 h at 4 °C before being subjected to size exclusion chromatography using a HiLoad 16/60 Superdex 200 pg column (GE Healthcare) equilibrated with 20 mM Tris, pH 8.0, and 300 mM NaCl. Gel-filtration fractions containing the PD-L1/durvalumab Fab complex was concentrated to 6 mg ml^{-1} in 20 mM Tris, pH 8.0, and 300 mM NaCl. Crystals of the complex were grown using hanging-drop vapor diffusion with a reservoir solution containing 0.1M Bis-Tris, pH 5.5, 25% PEG3350, 0.2 M ammonium sulfate at 20 °C within 10 days. Crystals were cryoprotected by brief immersion in well solution supplemented with 20% ethylene glycol, and flash frozen in liquid nitrogen. X-ray diffraction data were collected at 100 K on beamline 7A of the Pohang Light Source (PLS), Republic of Korea. The crystals belonged to space group $P2_12_12_1$ (a = 39.95, b = 97.40, c = 153.56 Å) and X-ray diffraction data were collected to a resolution of 2.65 Å, integrated and scaled using HKL2000 (HKL Research). The structure was solved by molecular replacement using Phaser with the separate structures of the Fab fragment and PD-L1 in the structure of PD-L1/BMS-936559 Fab complex (PDB code 5GGT) as search models. Due to the intrinsic elbow flexibility of a Fab fragment, the Fv region and the other region including C_{H1} and C_L domain were separated when used as a search model. At this point, the electron density corresponding the PD-L1/durvalumab Fab complex was prominent. Iterative rounds of refinement were done using PHENIX with manual inspection using COOT. Statistics for data collection and refinement can be found in Table 1. All structural figures were generated using Pymol (http://www.pymol.org).

SUPPLEMENTARY REFERENCES

1. Otwinowski, Z. & Minor, W. Processing of X-ray diffraction data collected in oscillation mode. *Method Enzymol.* **276**, 307-326 (1997).

2. McCoy, A. J. *et al*. Phaser crystallographic software. *J. Appl. Crystallogr.* **40**, 658–674 (2007)

3. Adams, P. D. *et al*. PHENIX: a comprehensive Python-based system for macromolecular structure solution. *Acta Crystallogr. D* **66**, 213–221 (2010)

4. Emsley, P. & Cowtan, K. Coot: model-building tools for molecular graphics. *Acta Crystallogr. D* **60**, 2126–2132 (2004)
